# Regulation of human cerebro-microvascular endothelial baso-lateral adhesion and barrier function by S1P through dual involvement of S1P_1_ and S1P_2_ receptors

**DOI:** 10.1038/srep19814

**Published:** 2016-01-27

**Authors:** Rachael Wiltshire, Vicky Nelson, Dan Ting Kho, Catherine E. Angel, Simon J. O’Carroll, E. Scott Graham

**Affiliations:** 1 Centre for Brain Research, University of Auckland, New Zealand; 2 Department of Pharmacology and Clinical Pharmacology, Faculty of Medical and Health Sciences, University of Auckland, New Zealand; 3 School of Biological Sciences, Faculty of Science, University of Auckland New Zealand; 4 Department of Anatomy, School of Medical Sciences, University of Auckland New Zealand

## Abstract

Herein we show that S1P rapidly and acutely reduces the focal adhesion strength and barrier tightness of brain endothelial cells. xCELLigence biosensor technology was used to measure focal adhesion, which was reduced by S1P acutely and this response was mediated through both S1P_1_ and S1P_2_ receptors. S1P increased secretion of several pro-inflammatory mediators from brain endothelial cells. However, the magnitude of this response was small in comparison to that mediated by TNFα or IL-1β. Furthermore, S1P did not significantly increase cell-surface expression of any key cell adhesion molecules involved in leukocyte recruitment, included ICAM-1 and VCAM-1. Finally, we reveal that S1P acutely and dynamically regulates microvascular endothelial barrier tightness in a manner consistent with regulated rapid opening followed by closing and strengthening of the barrier. We hypothesise that the role of the S1P receptors in this process is not to cause barrier dysfunction, but is related to controlled opening of the endothelial junctions. This was revealed using real-time measurement of barrier integrity using ECIS ZΘ TEER technology and endothelial viability using xCELLigence technology. Finally, we show that these responses do not occur simply though the pharmacology of a single S1P receptor but involves coordinated action of S1P_1_ and S1P_2_ receptors.

Sphingosine-1-phosphate (S1P) is a small bioactive lipid, which belongs to the lysophospholipid family^1^. S1P is produced by platelets, immune cells (mast cells), endothelial cells, and recent evidence has emerged for its production by astrocytes^1^,^2^. S1P mediates a range of cellular responses including; proliferation, cytoskeletal organization and migration, cell adhesion, and cellular junction assembly^3^. Lipoprotein bound S1P is present at approximately 200–900 nM in the blood and at much lower concentrations in peripheral tissues and cell types^1^,^4^,^5^. The production of S1P in tissues is governed by the coordination of two sphingosine kinases (SphK1 and 2). The maintenance of S1P levels is governed by the coordination of SphK1 and 2 and a combination of S1P phosphatase and lyase^1^,^6^. S1P is an agonist at 5 different receptors, which are all G-protein coupled receptors, named S1P receptors 1–5^7^ and henceforth referred to as S1P_1_ for receptor 1 and so on. The receptors for S1P are differentially and widely expressed throughout the body, and couple to different G proteins to activate differential signalling pathways^7^.

The therapeutic manipulation of the S1P pathway via the use of receptor agonists and antagonists has been a recent focus of the literature^1^,^8^. This is in part due to the FDA-approval (2010) of Fingolimod (FTY720; marketed as Gilenya) for the treatment of relapsing remitting multiple sclerosis. Fingolimod is believed to target the S1P receptors of secondary lymphoid tissues, preventing the egress of T cells from lymphoid tissues back to the vascular circulation^1^. This reduces the number of circulating lymphocytes and thus prevents the trafficking of autoimmune cells into CNS lesions across the blood brain barrier^8^.

Expression of some S1P receptors have been detected in the central nervous system (CNS) of various species. In human post-mortem brain tissue specimens the S1P_1_^9^,^10^,^11^,^12^, S1P_2_^13^ and S1P_5_^13^,^14^ isoforms have been shown to be expressed by the neurovascular unit using histological techniques. At least some of this expression appears to be by astrocytes^12^ in their end feet and some expression is by the endothelium^15^. We propose it is likely that different vessel types or brain regions express a different profile of S1P receptor expression, which would influence the nature of the response to S1P *in vivo*. This has not been studied in detail in the human brain and warrants further investigation.

*In vitro* studies have revealed differential effects of S1P on systemic vascular endothelium, due to binding at different receptor types. S1P binding to S1P_1_ has been shown to provide barrier enhancement and increases the electrical resistance of endothelial barriers^1^. Barrier enhancement has been observed to correlate with S1P_1_ activation of the G1/o-Rac pathway^16^. In contrast, S1P binding of S1P_3_ results in barrier disruption and loss of tight junction (TJ) formation^17^. The above data has historically been obtained utilising murine endothelial cells or endothelial cells from non-CNS tissues. Very recently the role of S1P_5_ at the BBB (blood brain barrier) endothelium has been investigated using the human hCMVEC/D3 BBB endothelial model^18^. Silencing of S1P_5_ resulted in the reduction of leukocyte migration and consequently less barrier disruption^18^. This suggests S1P binding to S1P_5_ may induce barrier disruption and increased permeability, particularly during inflammation.

Currently, the literature and research field concerning the role of S1P and S1P receptors of the human BBB vasculature is only beginning to emerge. While studies have identified the expression of S1P receptors in the brain vasculature using human post-mortem tissues, the effects of S1P on brain endothelial cells has not been analysed extensively^18^. This is due in part to the lack of human endothelial lines from the CNS and the innate difficulty of obtaining primary human microvascular cultures.

Based on studies using peripheral endothelial cells^16^,^17^ we hypothesised that there was a high probability that brain endothelial cells would be regulated by S1P, potentially in a manner that influences barrier function or barrier integrity. We utilised a new human brain microvascular endothelial model (hCMVECs), which we extensively characterised previously^19^. Here, we investigated the regulation of hCMVECs by S1P using a combination of cutting-edge technologies and selective receptor antagonists to reveal involvement of specific receptors. Initially, xCELLigence biosensor technology was used to monitor the window of S1P responsiveness and revealed that S1P reduced endothelial adhesion rapidly and acutely. S1P also increased secretion of several inflammatory cytokines and chemokines and increased surface expression of ICAM-1, albeit to a very low level. These responses are consistent with promoting localised endothelial-leukocyte communication but are not consistent with global inflammation. Electric cell-substrate impedance sensing (ECIS ZΘ) technology revealed that S1P mediated the rapid opening of the endothelial barrier, which was followed by the strengthening of the barrier integrity. This effect involved both S1P_1_ and S1P_2_. The response to S1P was neither cytotoxic nor consistent with causing barrier dysfunction as suggested in other studies. In this human microvascular endothelial model derived from the human brain, S1P is implicated in the controlled gating of endothelial barrier integrity in a manner consistent with barrier opening potentially for regulating entry of leukocytes.

## Results

### S1P rapidly and acutely alters the focal adhesion of brain endothelial cells

**Figure 1:**
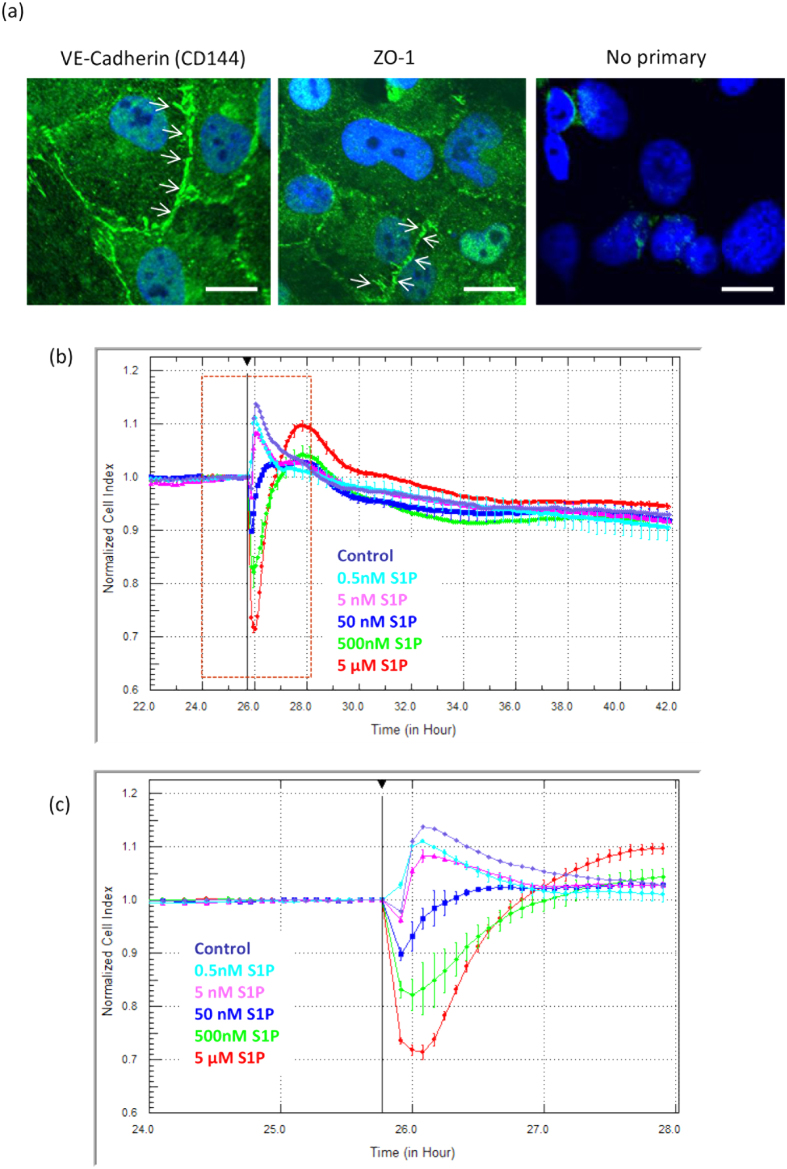
S1P rapidly and acutely alters brain endothelial focal adhesion. The hCMVEC monolayers form tight and adherens junctions that are evident 24 hours after seeding. (**a**) shows the expression of the junctional proteins VE cadherin (CD144) and Zonula Occludin-1 (ZO-1) 24 hours after seeding of cells to demonstrate formation of junctional complexes. The no primary control stain is also shown for comparison. (**b**) S1P affects the focal adhesion of hCMVECs in a concentration and time-dependent manner. S1P was added at the time demarcated by the black line. (**b**) reveals the gross effect of S1P on hCMVEC adhesion during the 16h period following S1P addition. (**c**) reveals the rapid response to S1P during the first few hours after addition. These data are representative of 6 independent observations. Note these data are normalised to the Cell Index at the time of S1P addition. See supplemental figure 1 online for the actual Cell Index values (not normalised) and full time course data.

**Figure 2:**
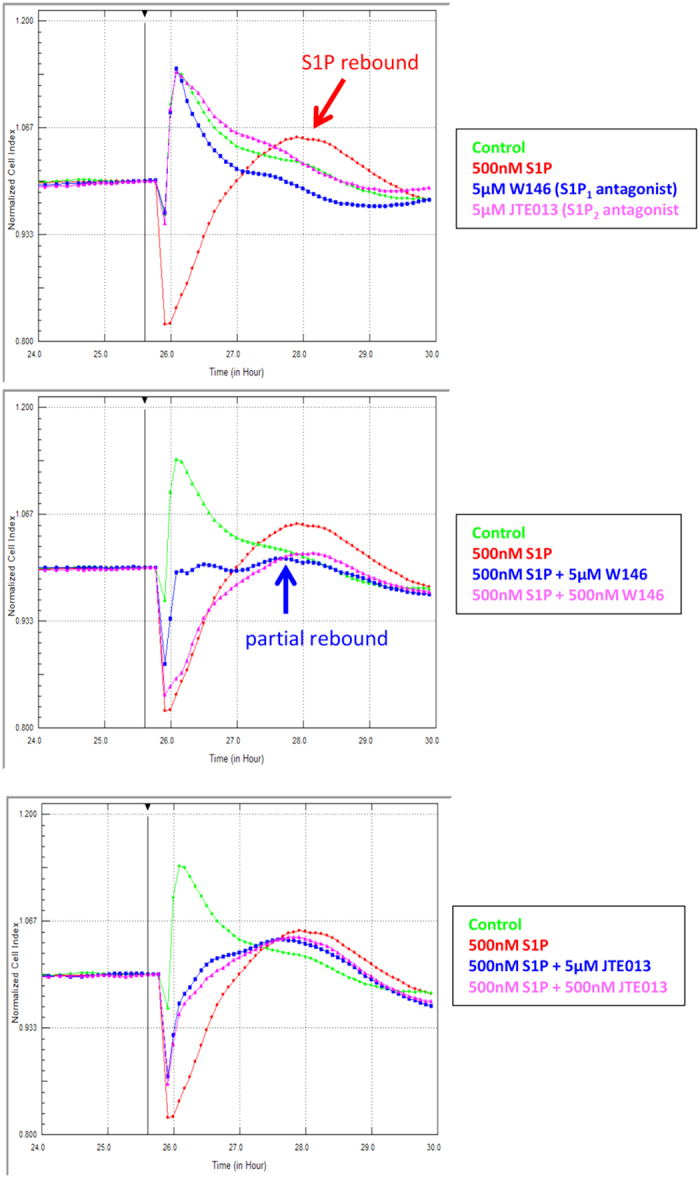
The reduction in focal adhesion induced by S1P requires both S1P_1_ and S1P_2_ receptors. Receptor specific antagonists were used to ascertain the involvement of each respective receptor in the S1P (500 nM) response. (**a**) There were no effects of the antagonists (5 μM) on basal focal adhesion. (**b**) The S1P_1_ antagonist (W146) partially blocked the effect of S1P. (**c**) The S1P_2_ antagonist (JTE013) also partially blocked the effect of S1P. Antagonists for S1P_3_ and S1P_4_ receptors had no effect on the S1P response (data not shown). These responses have been observed in 4 independent experiments.

We used xCELLigence RTCA biosensor technology to profile the temporal response of brain endothelial cells to various concentrations of S1P. This was conducted to better understand the window of responsiveness and pharmacology of the response. The hCMVEC endothelial cells achieve a stable, strong level of cellular adhesion (Cell Index >7) within ~24 hours of culture post seeding (supplemental Fig. 1). This represents the period where the cells form a visible monolayer, with formation of tight junctions as indicated by the Zonula Occludin-1 (ZO-1) and VE-cadherin staining (Fig. 1a). The addition of S1P caused an immediate reduction (within minutes) in baso-lateral (focal) adhesion, which was maximal ~10–15 minutes after S1P addition. This reduction was evident with S1P at 50 nM to 5 μM. After this reduction in focal adhesion, it required several hours for the Cell Index to rebound back to control levels (Fig. 1b,c, supplemental Fig. 1 online). S1P concentrations at 500 nM and 5μM typically mediated a slight increase in the focal adhesion following this rebound (see Figs 1 and 2).

Beyond this acute response there is no obvious long term effect on endothelial adhesion (supplemental Fig. 1 online), which indicated that the S1P is not cytotoxic, nor does it induce cellular proliferation. Pharmacological response profiling using xCELLigence RTCA biosensor technology is now well characterised^20^,^21^ and is a very important tool for providing the window of cellular responsiveness^20^,^22^.

As there are 5 different S1P receptors and all except S1P_4_ have been identified in the brain, we were curious as to which receptors mediated the changes in endothelial adhesion. Therefore, the commercially available highly selective antagonists to S1P_1_, S1P_2_, S1P_3_, and S1P_4_ (no commercially available S1P_5_ antagonists available) were obtained, and assessed to identify which S1P receptor mediated the response. Surprisingly, two antagonists attenuated the S1P response (Fig. 2b,c, and supplemental Fig. 2 online). These were W146, which is highly selective for S1P_1_ (no activity at S1P2, S1P3, or S1P5 receptors up 10 μM) and JTE013, which is highly selective for S1P_2_. JTE013 has no activity at S1P_1_ (JTE013 >10 μM). As expected, the antagonists for S1P_3_ and S1P_4_ had no effect basally or on the S1P response (data not shown). The S1P_1_ and S1P_2_ antagonists did not have any major effect on endothelial adhesion when given alone (Fig. 2a). However, both caused an immediate but partial block of the S1P mediated reduction in baso-lateral adhesion. This occurred at a faster rate and to a greater extent with the S1P_1_ antagonist. Note also that the S1P_1_ antagonist also reduced the rebound increase which is evident with S1P alone. The S1P_2_ antagonist also had an immediate effect on the S1P mediated reduction in baso-lateral adhesion but less of an effect on the rebound phase of the S1P response. These data implicate involvement of both S1P_1_ and S1P_2_ in the brain microvascular endothelial response to S1P. This is a highly novel finding suggesting the coordinated involvement of both receptors. We highlight the power of xCELLigence technology in realisation of these responses.

### S1P increases secretion of inflammatory cytokine and chemokine secretion

**Figure 3:**
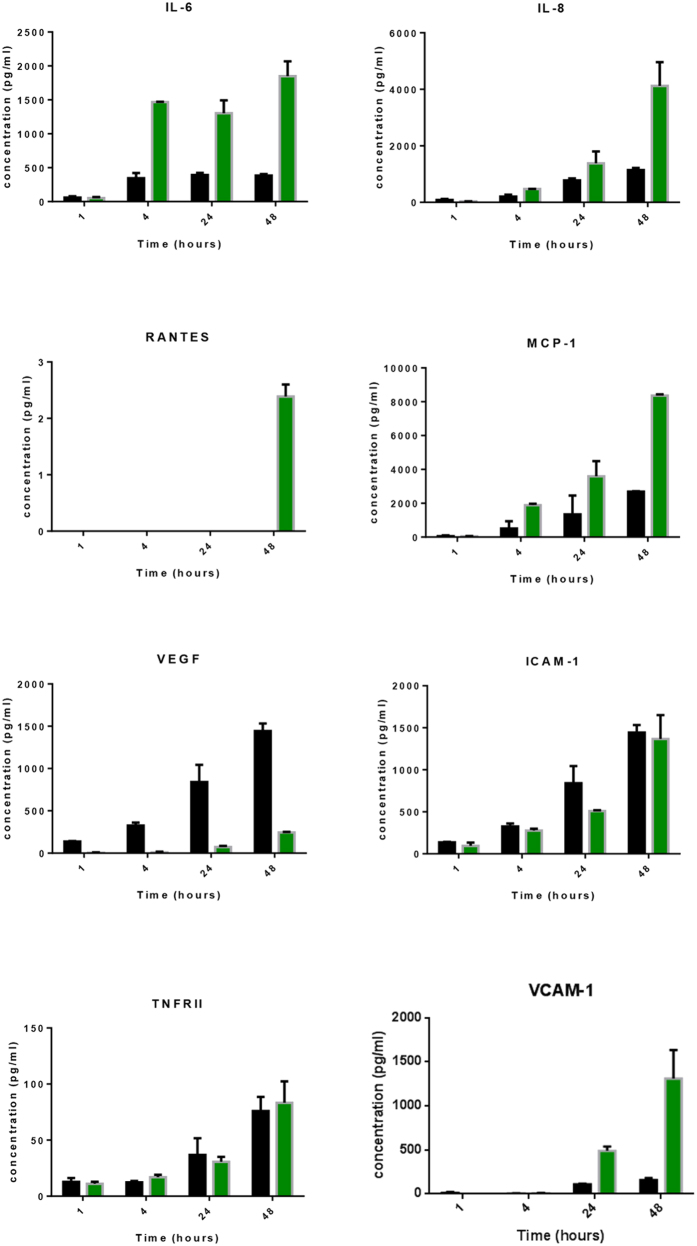
S1P increases secretion of specific inflammatory mediators. Secreted cytokines and soluble surface adhesion molecules were measured using multiplex cytometric bead array technology. Conditioned media was collected 1h, 4 h, 24 h and 48 hours after treatment with 500 nM S1P (green bars). The secretions from the vehicle treated cells (0.005% DMSO) are the black bars. Data show the mean ± SEM from 3 independent experiments.

We have recently conducted a comprehensive analysis of cytokine secretion by the hCMVEC endothelial cells^19^ using cytometric bead array (CBA) technology^23^,^24^,^25^. This original study demonstrated that the hCMVECs secreted a highly select range of cytokines and chemokines under inflammatory conditions including IL6, RANTES, MCP-1, VEGF, soluble ICAM-1 (sICAM-1), soluble VCAM-1 (sVCAM-1) and the soluble version of both TNF receptors. We therefore investigated the effect of 500 nM S1P on this panel of cytokines over a time course of 1 h, 4 h, 24 h and 48 h. These time-points were chosen from the xCELLigence response profile to cover both acute and longer term secretion. The temporal dynamics of S1P effects on cytokine secretion were different between the cytokines (Fig. 3). For example, IL-6 secretion was induced by S1P as early as 4 hours, which is consistent with the production of primary danger cytokines. Whereas, the chemokines IL-8 and MCP-1 were marginally elevated by S1P at 4–24 hours. However, 48 h after S1P addition, these chemokines were substantially elevated in comparison to control treated cells. RANTES was only detected at 48 h following S1P treatment, but these levels were very low (1–10 pg/mL). In contrast, S1P reduced the basal secretion of VEGF (see Fig. 3) by about 80–90%. Although there is an obvious influence of S1P on cytokine secretion, the amounts secreted in response to S1P are small in comparison to the responses to the pro-inflammatory cytokines TNFα and IL-1β (see supplemental Fig. 3 online).

The soluble versions of ICAM-1, VCAM-1 and the TNF receptors were also measured using CBA. The concentrations of sICAM-1, TNFRI and TNFRII were not greatly affected by S1P treatment at any time point in our study. Whereas, the concentration of soluble VCAM-1 was substantially increased at both 24 h and 48 h after S1P addition. It is unclear whether this represents actual secretion of a truncated version of this adhesion molecule or cleavage of cell surface expressed adhesion molecule.

### S1P increases cell surface expression of ICAM-1 but not VCAM-1

**Figure 4:**
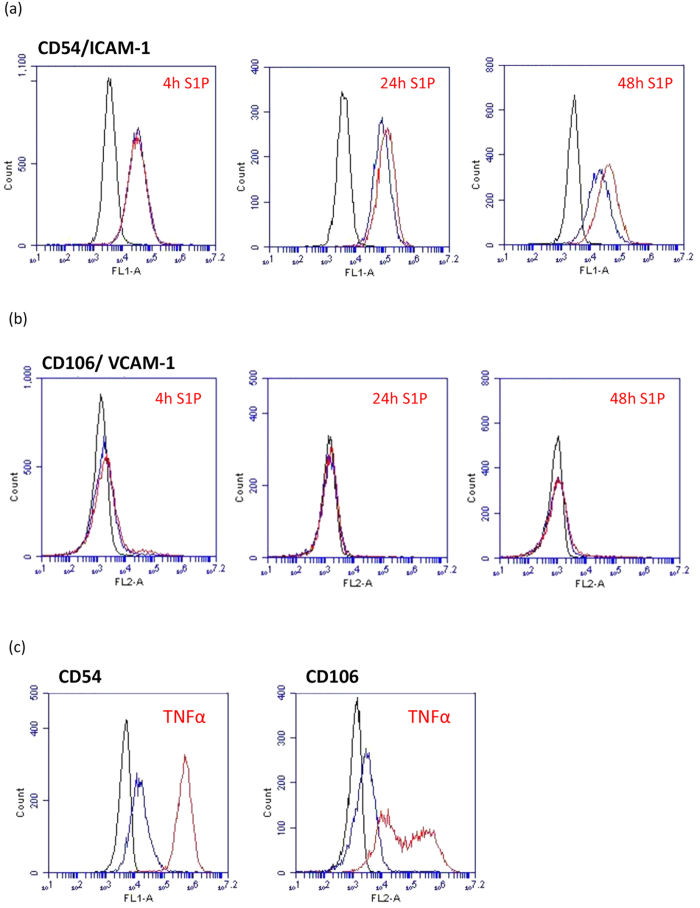
S1P regulation of key leukocyte tethering/adhesion molecules. The surface expression of (**a**) ICAM-1 and (**b**) VCAM-1 were measured by flow cytometry 4 h, 24 h and 48 h after treatment with 500 nM S1P. Panels in (**c**) show the pronounced effect of the potent pro-inflammatory mediator TNFα on CD54/ICAM-1 and CD106/VCAM-1 expression by the hCMVEC as a comparison. In each histogram the black population is the background cellular auto fluorescence, the blue curve is the basal ICAM-1 or VCAM-1 expression and the red curve is the expression following treatment with S1P (a + b) or TNFα (**c**). These responses have been observed in 3 independent experiments.

**Figure 5:**
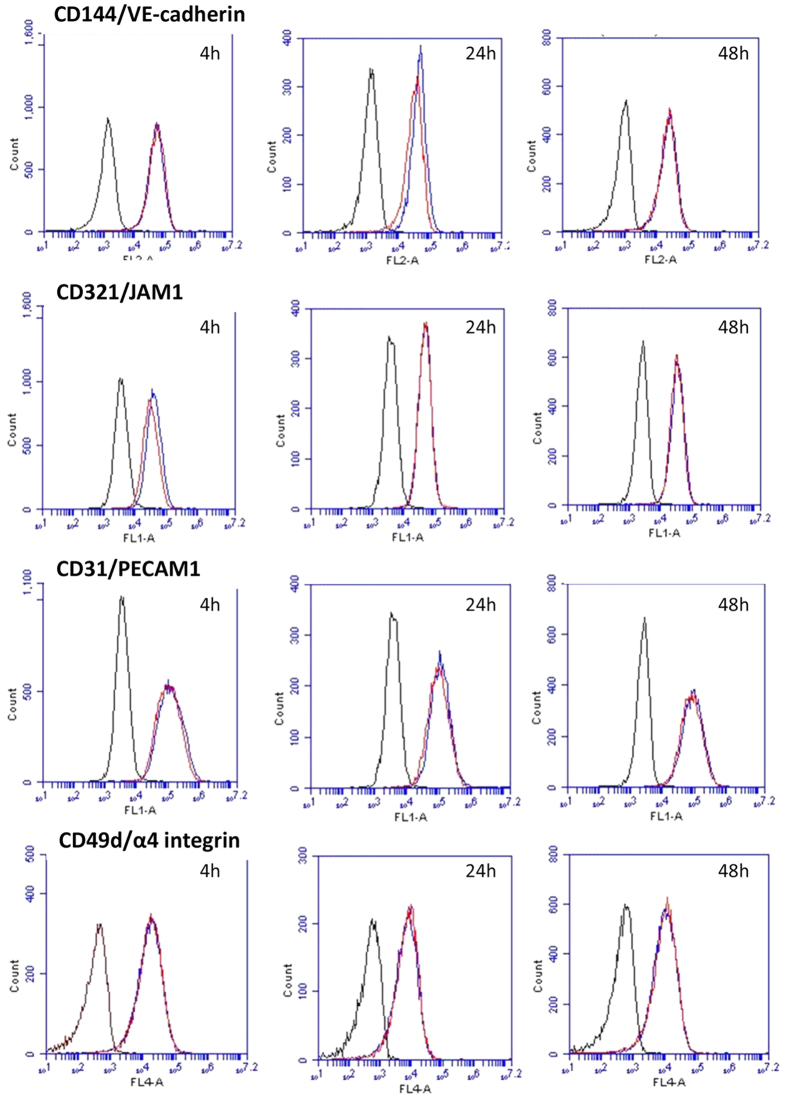
S1P has no gross effect on the expression level of other key cell surface endothelial adhesion molecules. Cell surface expression of CD144, CD321, CD31, CD49d were measured 4 h, 24 h and 48 hours after treatment with 500 nM S1P. Each cell adhesion molecule is expressed by the hCMVECs. In each histogram the black population is the background cellular auto fluorescence, the blue curve is the basal expression (CD144/CD321/CD31/CD49d) and the red curve is the expression following treatment with S1P (500 nM). These responses have been observed in 3 independent experiments.

As sVCAM-1 levels were elevated in the hCMVEC conditioned media we hypothesised that S1P would induce a concordant increase in surface VCAM-1 expression. This was therefore measured using flow cytometry over a 48 h time course where ICAM and VCAM-1 would be expected to be induced following inflammatory activation of the endothelial cells^19^. However, S1P treatment did not increase surface VCAM-1 expression but did increase surface ICAM-1 slightly, which was evident at both 24 h and 48 h after S1P addition. It should be noted that the influence of S1P on surface ICAM-1 was very small. In comparison, TNFα (a potent pro-inflammatory cytokine) up regulated both ICAM-1 and VCAM-1 (shown in Fig. 4c) more than 50 fold. In addition to these critical leukocyte tethering adhesion molecules we investigated CD144, CD321, CD31 and CD49d expression across the 4 to 48 hour time course (Fig. 5). None of these were affected by S1P treatment across this time period.

### S1P mediates acute opening of the endothelial barrier followed by closing and strengthening

**Figure 6:**
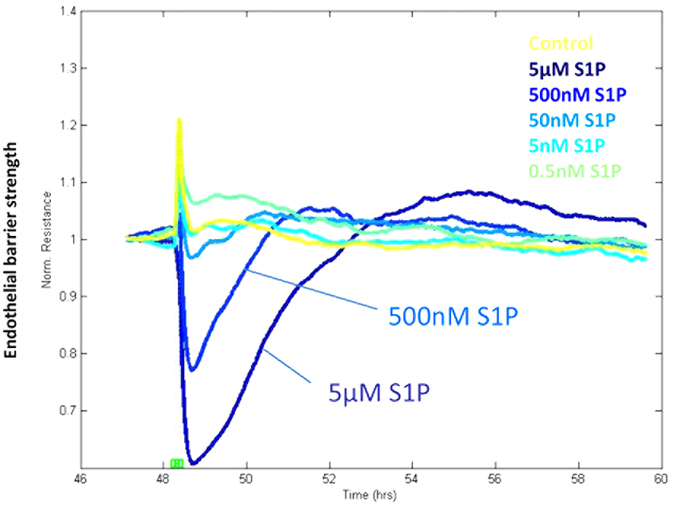
S1P rapidly and acutely reduces the trans-endothelial electrical resistance, which is followed by strengthening of the endothelial barrier. The temporal ECIS data shows rapid and acute reduction in endothelial barrier resistance following S1P addition (dotted lines) at 500 nM and 5 μM. This is followed by slower strengthening of the barrier resistance (3–4 hours) resulting in a stronger barrier resistance than before S1P treatment. Data show mean ± SD (4 wells). These responses have been observed in 6 independent ECIS experiments.

The changes observed in cytokine secretion and adhesion molecule expression are consistent with the microvascular endothelial cells responding to S1P in a manner involving leukocyte communication or low level recruitment. Such events at the blood-brain barrier are often associated with acute changes in endothelial barrier function. We used ECIS ZΘ impedance based trans-endothelial electrical resistance (TEER) technology^26^,^19^ to measure the real time changes in the hCMVEC barrier activity following S1P treatment. The hCMVEC monolayer resistance (ohms; Ω) was measured in real time initially to ascertain when a stable barrier was formed. Typically, this occurred 20–30 hours after seeding (see supplemental Fig. 4 online) and coincides with the formation of abundant junctional adhesion molecules (see Fig. 1a). The hCMVECs achieved a basal TEER level of 800 to 1200Ω when using the high density ECIS ZΘ arrays (measured at 4000 Hz). This level of TEER, indicative of a strong barrier, was typically retained for >72 hours. Therefore, the cells were stimulated with S1P ~48 h after seeding during the window of the highest TEER (tightest barrier under basal conditions). ECIS revealed that S1P caused an immediate and very transient reduction in barrier resistance (see Fig. 6). Again this effect on barrier resistance was evident with S1P at 50 nm to 5 μM (inclusive) and was concentration dependent. The reduction in endothelial barrier strength was very transient (5–10 minutes) and after barrier opening, the barrier resistance increased but at a slower rate, requiring 2–4 hours to return to the control barrier level. At the higher concentrations of S1P (500 nM and 5 μM), there was a rebound (~15%) above the control barrier level, which is indicative of barrier strengthening. The level of this rebound varied between experiments but was always observed. This response is consistent with S1P mediating rapid but controlled opening of the barrier, followed by closing and strengthening of the barrier (increase in TEER).

**Figure 7:**
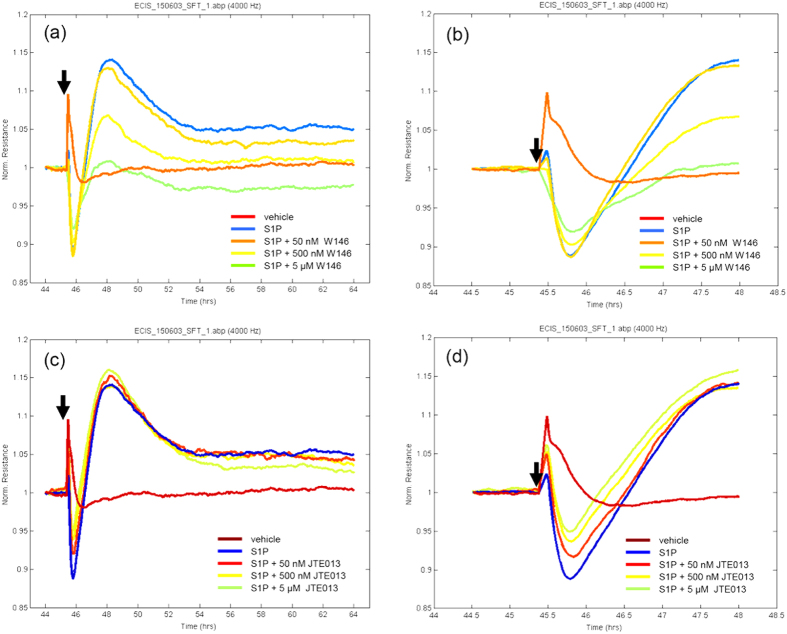
S1P induced changes in barrier function mediated through both S1P_1_ and S1P_2_ receptors. The barrier strength of the hCMVECs measured using ECIS. Normalised TEER of 1 is equivalent to ~900Ω. (**a,b**) influence of the S1P_1_ antagonist W146 (50 nM to 5 μM) on the S1P response. (**c,d**) influence of the S1P_2_ antagonist JTE 013 (50 nM to 5 μM) on the S1P response. The (**b**) and (**d**) panels focus on the 3 h period following drug addition, to highlight the acute effects of the antagonists. Data are normalised to the time of drug addition. Similar responses were obtained in 3 independent experiments.

Given the intriguing involvement of both S1P_1_ and S1P_2_ on baso-lateral adhesion of the hCMVEC (Figs 1 and 2), it was an obvious next step to ascertain which receptors were involved in the acute changes in barrier strength. The S1P_1_ antagonist had a partial effect on the barrier opening response, and a substantial effect on the magnitude of the rebound. This effect on the barrier strengthening (rebound) is clearly evident in Fig. 7a,b and occurred in a concentration dependent manner. Conversely, the S1P_2_ antagonist had very little effect on the rebound response but partially blocked the opening phase induced by S1P. This can be observed in Fig. 7c,d, particularly in the right hand panel, which highlights the acute barrier opening response. Taken together it is clear that S1P has a direct effect on the basolateral focal adhesion (xCELLigence data; Figs 1 and 2) and also influences the strength of the endothelial barrier (ECIS data).

## Discussion

### Executive summary

The aim of this study was to ascertain whether S1P regulates cerebral microvascular endothelial cells in a context that would lead to alteration of barrier integrity (weakening or strengthening). The rationale for this is that S1P receptors are expressed in the human brain (S1P_1_^9^,^10^,^11^,^12^, S1P_2_^13^ and S1P_5_^13^,^14^) and previous literature suggests S1P involvement in controlling vascular function in peripheral tissues (reviewed by^1^). Initially we used xCELLigence RTCA Biosensor technology as prior experience demonstrated its power to reveal global responses and the temporal window (e.g. rapid responses) of responsiveness^20^,^21^. xCELLigence revealed that the endothelial response to S1P was very fast, resulting in a change in endothelial morphology or adhesion. xCELLigence technology sensed this within minutes of the S1P being added to the cells. ECIS TEER measurements revealed that S1P also acutely reduced endothelial barrier resistance, which was followed by closing and strengthening of the barrier. The influence of S1P on endothelial adhesion and barrier function involved S1P_1_ and S1P_2_ receptors. This is a highly novel and complex dynamic, where cooperation of both receptors is suggested based on the antagonist data.

The rapid rate of the response leading to such a substantial change in endothelial adhesion was very informative for all downstream experiments. The temporal xCELLigence data helped define the time course used for the cytokine measurements. This translated to measuring cytokine levels at 1 hour and 4 hours after S1P addition. These time-points were valuable as IL-6, VEGF, MCP-1 IL-8 and soluble ICAM-1 and TNFRII were all detectable at low levels at these time points. Importantly S1P caused an increase in IL-6, IL-8 and MCP-1, which was evident as early as 4 hours, whereas secretion of VEGF was substantially reduced at all time-points including the 1 to 4 hour responses. Basal secretion of all of the inflammation-related cytokines was very low, which is consistent with healthy non-inflamed cells. S1P clearly increases the secretion of IL-6, MCP-1 and IL-8. However, potent pro-inflammatory mediators, like IL-1β or TNFα, increase secretion of these factors to a much greater extent (see supplemental Fig. 3 online). This is a very interesting comparison as it suggested that S1P may cause vascular activation but at much lower or more localised level. In addition, potent pro-inflammatory mediators (e.g. TNFα/IL-1β) typically induce an increase in the expression of surface ICAM-1 and VCAM-1^19^, which are key mediators of leukocyte attachment and extravasation across the brain vasculature during neuroinflammation. Whereas S1P did not increase surface expression of VCAM-1 and only increased ICAM-1 expression slightly. Intriguingly, there was an increase in soluble VCAM but no increase in surface VCAM. This suggests that the origin of the sVCAM was from secretion rather than cleavage. In this context, secretion of sVCAM may act as a decoy and reduce local leukocyte adhesion. Collectively, these observations are not consistent with S1P having a global pro-inflammatory role at the brain endothelium. Rather they are suggestive of a much more subtle or localised response by the endothelial cells.

Next we investigated if S1P affected the tightness or integrity of the endothelial barrier. We already knew that S1P rapidly activated the endothelial cells. Therefore, in order to measure TEER over such an acute time frame we used an automated TEER system equipped with a 96-well platform (ECIS ZΘ) for this purpose^27^,^28^,^29^. Our previous experience using a manual EVOM Ω TEER meter (hand-held) demonstrated that it was very challenging to measure acute (minute time frames) responses reliably or accurately (data not shown). In contrast, ECIS ZΘ TEER technology measures the electrical resistance (Ω; ohms) across the endothelial layer continuously in real-time^30^. This was essential as S1P reduced endothelial TEER within minutes and the opening of the barrier was maximal by 10–20 minutes after S1P addition. The reduction in TEER was very fast and transient. The magnitude of the response was also relatively small (5% to 25% reduction in TEER). Interestingly, and perhaps very importantly we observed that there was a rebound above the control TEER level within 3–4 hours, which represented barrier strengthening of approx. 15–20%. ECIS has also been used to measure the effects of S1P on endothelial and epithelial cells of non-brain origin^31^. S1P increased the barrier strength of rabbit corneal endothelial cells but had no effect on retinal epithelial barrier resistance. The profile of the changes to the barrier strength was quite distinct from that observed here and the receptors involved in the response were not identified^31^. Brain endothelial expressed S1P_1_ has also been implicated in the protection of the BBB by protein S during hypoxic-ischemic injury^32^. In this elegant study, the authors demonstrated a novel association between the protein S receptor (Tyro3) and S1P_1_, which collectively functioned to prevent hypoxia induced BBB breakdown^32^.

Collectively, the changes in barrier tightness mediated by S1P are consistent with regulated opening of the barrier followed by controlled closing and strengthening. The tightness and integrity of the brain vasculature is governed at the molecular level by tight-junctions and adherens junctions^33^, which form between opposing endothelial cells thus sealing the vessels. We predict that an acute rearrangement or dissociation of the tight-junction or adherens junction complexes could explain the rapid reduction in TEER mediated by S1P within this time frame. Such a small difference in barrier resistance will be challenging to visualise at the molecular level but this will be investigated in a future study.

Finally, we reveal a highly novel cooperative involvement of both S1P_1_ and S1P_2_ receptors in brain endothelial barrier function. With the time-resolved power of ECIS it has been possible to show that the initial barrier opening involved both S1P_1_ and S1P_2_, whereas the rebound phase (strengthening) is primarily mediated through S1P_1_. It is unclear at this stage whether the receptors are working as a signalling collaboration or whether they exist as receptor dimers. Our observations are in disagreement to that reported by van Doorn and colleagues^14^ who reported that S1P_5_ was involved in the barrier function of the hCMEC/D3 cell line. We suspect that this is due to the origins (e.g. brain region, vessel types, donor-specific variables (e.g. sex)) of the endothelial cells and differences in the expression profile of the receptors between the hCMEC/D3 cells and the hCMVECs. S1P_5_ expression was reported to be expressed in brain capillaries^14^, whereas others report that S1P_5_ is exclusively expressed by oligodendrocytes^12^,^34^,^35^. Once S1P_5_ specific antagonists become available, we can investigate the extent of its involvement in the hCMVEC responses to S1P. Future experiments will also decipher the molecules involved in the S1P response leading to the strengthening of the endothelial barrier.

### Future Considerations

Our data suggests that S1P regulates the barrier in a manner consistent with highly regulated controlled opening (e.g. as may occur during T-cell immune-surveillance of the CNS) and closing of the barrier structure. The S1P effects are not consistent with a global pro-inflammatory response and are also not consistent with barrier dysfunction (ECIS and xCELLigence data). Rather, we hypothesise that S1P may be the missing link in the controlled opening of the brain vasculature during leukocyte surveillance during steady-state homeostasis of the CNS. Within this proposition the leukocytes or the endothelial cells could be the source of the S1P, where-by the action of the S1P is the key to highly regulated opening of the barrier for a short period of time.

Finally, it is important to consider that the human brain is highly vascularised and has different types of vessels (i.e. large and small arteries, arterioles, and capillaries). We hypothesise that different vessel types or brain regions may have differential expression of S1P receptors and biosynthesis/degradation enzymes (phosphatases, kinases and lyase). Further research is required to fully map expression of the S1P system (e.g. receptors, biosynthesis enzymes and transporters/carrier proteins) and this is essential to fully understand the function and potential of S1P in the human brain.

## Methods

### S1P and S1P receptor antagonists

S1P was purchased from Tocris. S1P receptor antagonists were purchased from Sapphire Biosciences (SB) and Abacus ALS (AALS). These were W146 for S1P_1_ (SB, catalogue # 10009109). JTE013 for S1P_2_ (SB, catalogue # 10009458). Suramin for S1P_3_ (SB, catalogue # ALX-430-022-M050). CYM50358 for S1P4 (AALS, catalogue # MPCA5677375MG). Drugs were prepared following the manufacturer’s instructions. In all experiments using S1P and antagonists, the DMSO-diluent was also included in the control/vehicle treatment. The concentration of DMSO was kept below 0.05% in all experiments.

### Cell culture of hCMVECs

The hCMVEC cell line was purchased from ABMGood (USA). Cells were maintained in M199 media (Gibco) supplemented with 10% FBS, 1 μg/mL hydrocortisone (Sigma), 3 ng/mL human FGF (Peprotech), 10 ng/mL human EGF (Peprotech), 10 μg/mL heparin (Sigma), 2mM Glutamax (Gibco) and 80μM di-butyryl cAMP (Sigma); referred to as M199 10% growth media. The hCMVEC experiments were typically conducted using low serum plating media containing M199 media, 2% FBS, 110 nM hydrocortisone, 1 μM insulin and 80 μM butyryl cAMP (M199 2% plating media). In all experiments, cells were seeded at a density close to 100% confluence to achieve an endothelial monolayer and barrier as quickly as possible.

### Immunocytochemistry

For confocal imaging, the cells were grown to 100% confluence on collagen-coated 8-chamber glass slides (BD Biosciences. Cat, no: 354108). Cells were fixed for immunocytochemical staining using 4% PFA at room temperature for 10 min. Excess PFA was removed with several washes using PBS. Primary antibody against VE-cadherin/CD144 (Santa Cruz Biotechnology Inc, sc-6458) was prepared in PBS containing 1% donkey serum and used at 1:200 dilution. Primary antibody to zonula occludin-1 (ZO-1; Invitrogen, 339100) was prepared in PBS containing 1% goat serum and used at 1:500 dilution. Primary antibodies were incubated at 4 °C overnight. Unbound antibodies were removed by washing in PBS containing 0.2% triton-X100 (PBST). Fluorophore conjugated secondary detection was used using the species-specific anti-IgG Alexafluor 488 antibodies from Life Technologies (A-21206 and A11055). These secondary antibodies were used at 1:400 and incubated with cells for 2h at ambient. Again, unbound antibodies were removed with gentle agitation with PBST washes. Nuclei were counter stained using Hoescht (1:500 dilution in PBST), and cells were mounted using AF1 mounting media. Confocal imaging was conducted using an Olympus FV100 microscope. Images were merged using ImageJ software.

### xCELLigence experiments

The xCELLigence real time cell analyser (RTCA) measures focal adhesion of living cells in real-time. Cells are seeded onto custom RTCA E-plates, which are coated with high-density gold arrays for measuring electrical impedance (ACEA Biosciences, USA)^36^,^37^. The xCELLigence biosensor measures cellular adhesion, which is converted to Cell Index (unit less) by the xCELLigence software (version 1.2.1). Cells were seeded into E96 well plates (coated with 1 μg/cm^2^ collagen) at 54,000 cells per well in M199 2% plating media and allowed to recover until the cells had attained a stable Cell Index. This was typically 24 hours after seeding. Treatments are detailed in the corresponding figure legends.

### Stimulation of hCMVEC for cytokine production

The hCMVEC cultures were seeded into 24-well plates coated with 1 μg/cm^2^ collagen I (Gibco) at a density of 80,000 cells per well. Cells were grown to 100% confluence in M199 10% growth media. On the day of stimulation, the growth media was changed to M199 2% plating media. Stimulatory pro-inflammatory cytokines (IL-1β and TNFα) were added to corresponding wells to a final concentration of 5 ng/ml (from stock 50 μg/mL in PBS/0.1%BSA). The control group received media only. 100 μl of conditioned media was collected at 4h, 24h and 72h post-treatment. The collected media was centrifuged at 400× g for 5 min at 4 °C to remove cellular debris. 80 μl of the clarified media was recovered and stored at −20 °C in several single use aliquots for cytokine profiling.

### Cytokine measurements using Cytometric Bead Array (CBA)

Soluble cytokines in the hCMVEC conditioned media were measured simultaneously using multiplexed bead-based Cytometric Bead Array (CBA, BD Biosciences). The assay was conducted using 25 μl of sample and a 10 point standard curve (ranging from 0 to 5000 pg/ml) was included for each cytokine measured (see table 1 for list of cytokines). The samples were analysed using a BD Accuri C6 flow cytometer (BD Bioscience). FCAP Array software (BD version 3.1) was used to create the standard curves for each cytokine and convert the fluorescent MFI values into cytokine concentrations.

### Cell surface flow-cytometry analysis of hCMVEC

The hCMVEC cultures were seeded into T75 maintenance flasks and were grown to confluence in M199 10% growth media. On the day of stimulation, the media was changed to M199 2% plating media with 5 ng/ml IL-1β, 5 ng/ml TNFα (from stock 50 μg/mL in PBS/0.1%BSA) or no stimulatory cytokine added for control group. After the designated treatment period (see figure legends) the endothelial cells were carefully harvested with EDTA-based Versene (Gibco). The use of trypsin was avoided as it can cleave cell surface epitopes. Cell suspensions were adjusted to the concentration of 1 × 10^6^ cells/ml with cold FACS buffer (PBS and 1% FBS) in 100 μl per tube. Fluorochrome conjugated antibodies (see table 1 for list of antibodies) were added to the cells at previously optimised dilutions, and were incubated on ice for 10 min. Cells were washed twice with 1 ml of cold FACS buffer, and centrifuged at 400× g for 10 min. The supernatant was discarded and cells were re-suspended in approximately 100 μl of FACS buffer. Flow cytometry was conducted using an Accuri C6 flow cytometer (BD Bioscience) calibrated with appropriate compensation controls. Each staining combination was incubated with 7AAD for live-dead cell determination. 7AAD positive cells were ascribed to the dead gate (P2) and excluded from further analysis. 7AAD negative cells represent the viable population and were ascribed as the live-gate (P1) (as described previously^19^). The specific staining of the flow-antibodies (detailed in table 1) was measured for the live-gate P1 only.

### Barrier Integrity measurements using ECIS ZΘ TEER technology

ECIS experiments were conducted using 96W20idf ECIS arrays. Wells were prepared by coating with 10 mM cysteine followed by coating with 1 μg/mL collagen I giving 1 μg/cm^2^ (as per manufacturer’s recommendation). After collagen coating, the arrays were placed in the ECIS array station to be stabilised to ensure the electrodes were properly connected and were not damaged by the coating procedure.

Following array preparation, the hCMVECs were harvested from a confluent T75 flask using 0.5% trypsin-EDTA (Life Technologies; Gibco). Cells were used between P7 and P17 and seeded at 20,000 cells in 100 μL of M199 10% growth media. ECIS was conducted using the single frequency option at 4000Hz, which is the recommendation for measuring barrier resistance. Additionally, it was impractical to use the multi-frequency option for the S1P responses as this process took approx. 12 minutes to measure the entire 96 well plate.

Cells were typically treated around 48 hours after seeding, which was determined previously as the period where the TEER level had stabilised (800–1000Ω). Drugs were added to respective wells as 2x concentrates. The final concentration of DMSO (S1P vehicle) was controlled and kept below (0.05%). Treatments were conducted in at least triplicate. Following drug addition, the ECIS experiments were continuously monitored for 2–3 days to capture both acute and longer term changes in the TEER.

## Additional Information

**How to cite this article**: Wiltshire, R. *et al.* Regulation of human cerebro-microvascular endothelial baso-lateral adhesion and barrier function by S1P through dual involvement of S1P_1_ and S1P_2_ receptors. *Sci. Rep.* **6**, 19814; doi: 10.1038/srep19814 (2016).

## Supplementary Material

supplemental figures

## Figures and Tables

**Table 1:** Details of the CBA flex sets and flow-cytometry antibodies and reagents used in this study.

CBA Flex Set	catalogue number
Human soluble CD106 (VCAM-1) Flex Set	560427
Human soluble CD54 (ICAM-1) Flex Set	560269
Human Soluble TNFRI Flex Set	560156
Human Soluble TNFRII Flex Set	560155
Human IL-8 Flex Set	558277
Human IL-6 Flex Set	558276
Human MCP-1 Flex Set	558287
Human RANTES Flex Set	558324
Human VEGF Flex Set	558336
**Flow cytometry antibodies**	
CD31 Alexa 488	303110
CD34 APC	343608
CD49d APC	304308
CD54 FITC	353108
CD106 PE	305806
CD144 PE	348506
CD321 FITC	353503
7AAD (BD Biosciences)	51-68981E

All CBA sets were purchased from BD and all flow antibodies were purchased from BioLegend.
