# Supplementary material for: Regulation of human cerebro-microvascular endothelial baso-lateral adhesion and barrier function by S1P through dual involvement of S1P1 and S1P2 receptors
Source: Sci Rep. 2016 Jan 27;6:19814. doi: 10.1038/srep19814 (PMC4728386; doi:10.1038/srep19814)
Supplement: supplemental figures [file srep19814-s1.doc]

# Regulation of human cerebro-microvascular endothelial baso-lateral adhesion and barrier function by S1P through dual involvement of S1P_1_ and S1P_2_ receptors.

**Authors , Rachael Wiltshire^1^, Vicky Nelson^1,2^, Dan Ting Kho^1,2^, Catherine E Angel^3^, Simon J O’Carroll ^1,4#^ , E Scott Graham ^1,2#*^.**

**Affiliations**

**^1^**Centre for Brain Research, University of Auckland, New Zealand.

^2^Department of Pharmacology and Clinical Pharmacology, Faculty of Medical and Health Sciences, University of Auckland, New Zealand.

**^3^**School of Biological Sciences, Faculty of Science, University of Auckland New Zealand

^4^ Department of Anatomy, School of Medical Sciences, University of Auckland New Zealand

# share senior authorship.

***Address for corresponding Author**

Dr E Scott Graham

Head of NeuroImmune Interactions Research Group

Research; Centre for Brain Research (Office 503-501B)

Teaching; Department of Pharmacology and Clinical Pharmacology

School of Medical Sciences

Faculty of Medical and Health Sciences

University of Auckland

New Zealand

[s.graham@auckland.ac.nz](mailto:s.graham@auckland.ac.nz)

+64 9236947

**Supplemental Figure 1.**


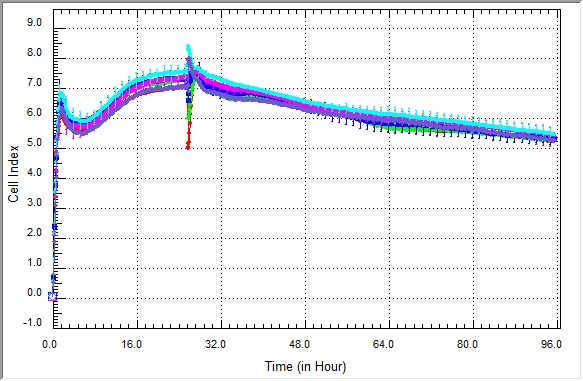

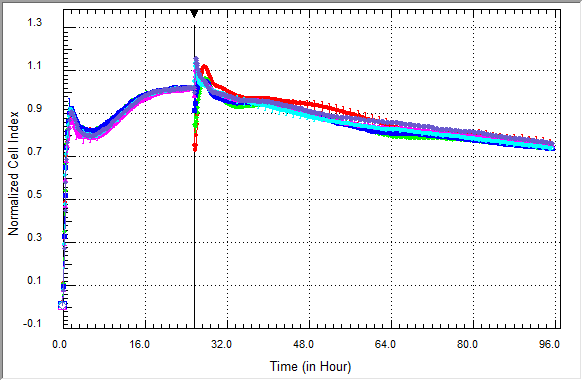

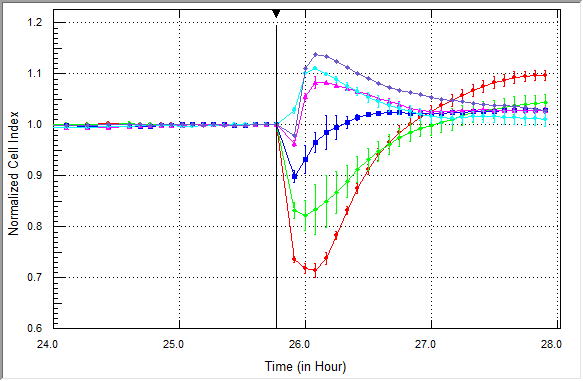

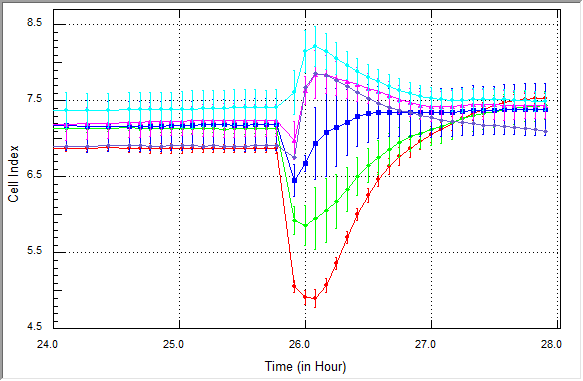


(a)

(b)

**Cell Index**

**Normalised Cell Index**

**Cell Index**

**Normalised Cell Index**

**Control**

**0.5nM S1P**

**5 nM S1P**

**50 nM S1P**

**500nM S1P**

**5 µM S1P**

**Control**

**0.5nM S1P**

**5 nM S1P**

**50 nM S1P**

**500nM S1P**

**5 µM S1P**

**Control**

**0.5nM S1P**

**5 nM S1P**

**50 nM S1P**

**500nM S1P**

**5 µM S1P**

**Control**

**0.5nM S1P**

**5 nM S1P**

**50 nM S1P**

**500nM S1P**

**5 µM S1P**


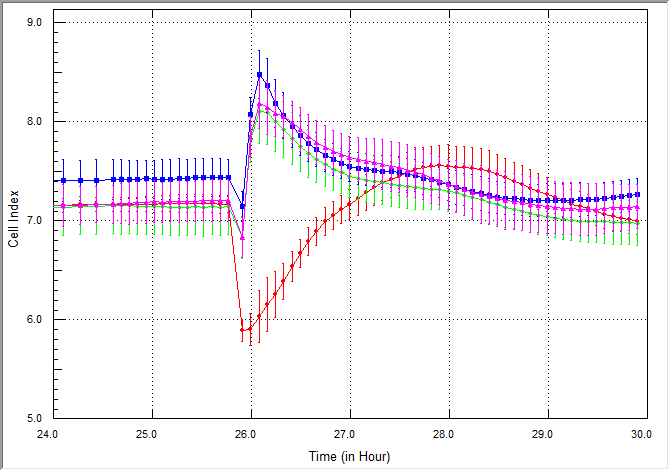


**Control**

**500nM S1P**

**5µM W146 (S1P_1_ antagonist)**

**5µM JTE013 (S1P_2_ antagonist**

**Supplemental Figure 2.**


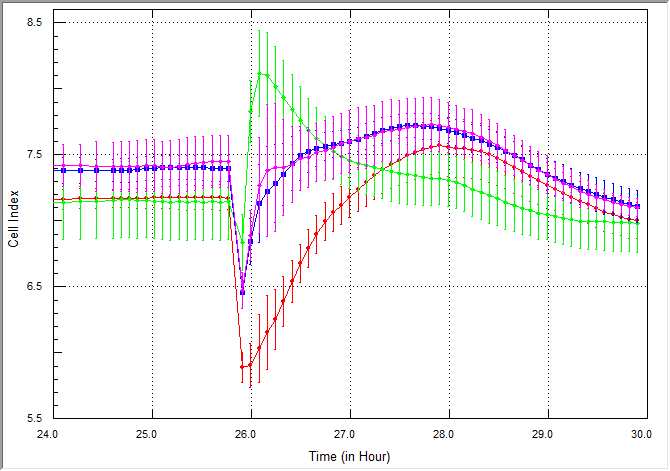


**Control**

**500nM S1P**

**500nM S1P + 5µM JTE013**

**500nM S1P + 500nM JTE013**


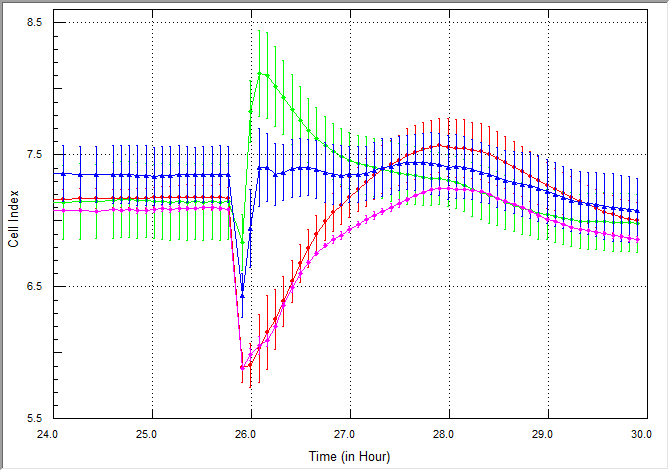


**Control**

**500nM S1P**

**500nM S1P + 5µM W146**

**500nM S1P + 500nM W146**

**Supplemental Figure 3.**


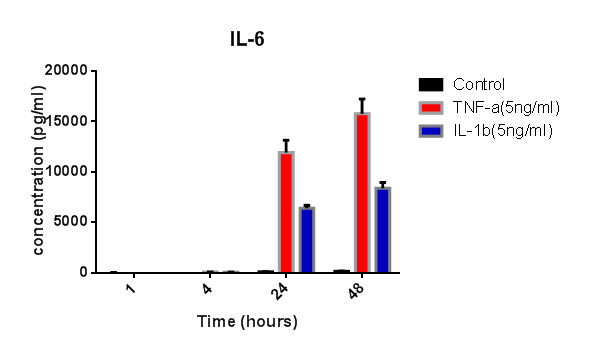

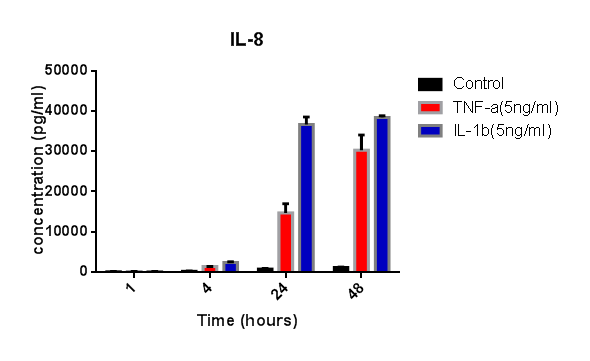

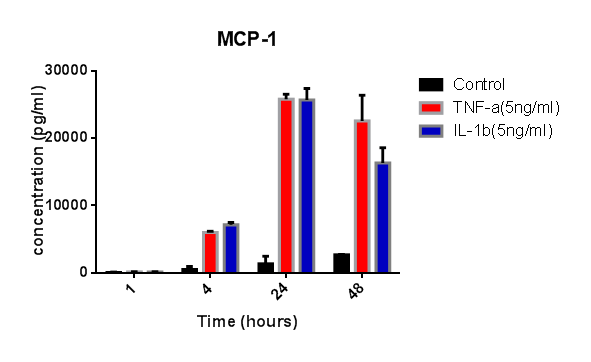

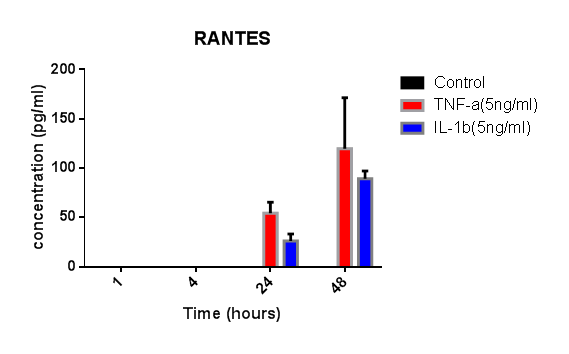


**Supplemental Figure 4.**


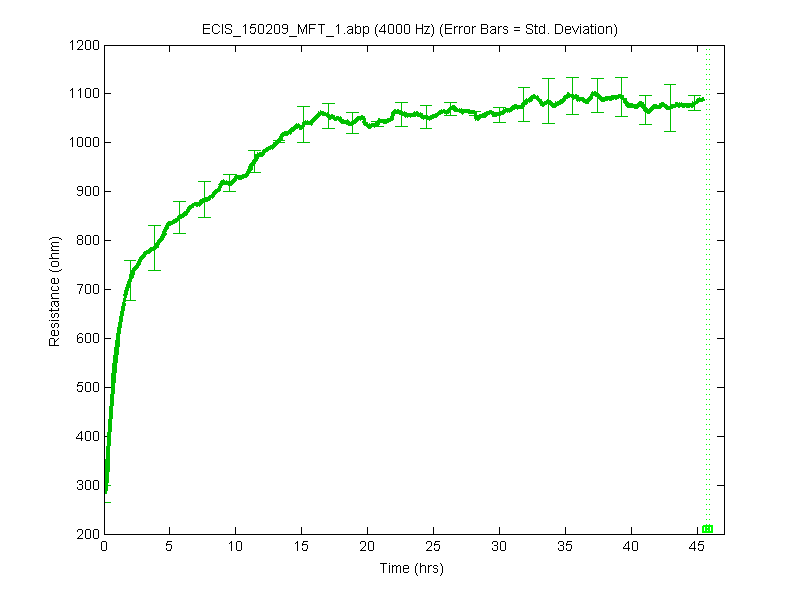


**Supplemental Figure 1. Full temporal xCELLigence RTCA Cell Index and Normalised Cell Index focal adhesion profiles.** (a) Cell Index profiles for the hCMVECs reveals the rapid acute response to S1P and that there is no evidence of a cytotoxic effect of S1P. Panels in (b) show the immediacy of the S1P response and the concentration-dependent effect. The raw Cell Index data reveals that the hCMVEC are strongly adherent with a Cell Index of 6-8. The normalised data reveals a maximum reduction in focal adhesion of ~28% with the highest concentration of S1P (5μM). Note that S1P at 5nM and 50nM reduced the adhesion by ~5% and 10% respectively. These responses are consistent with a more localised response rather than barrier dysfunction.

**Supplemental Figure 2. xCELLigence RTCA Cell Index antagonist responses.** These are the raw Cell Index values of the data shown in figure 2 (normalised). Data in each plot show the mean Cell Index (n=4 wells) ± SD.

**Supplemental Figure 3. Comparison of S1P, IL-1β and TNFα induced cytokine secretion.** S1P (500nM) increases secretion of IL-6, IL-8, MCP-1 and RANTES. In comparison, IL-1β and TNFα increase these cytokines 10-100 fold more than that induced by S1P. This highlights that S1P is substantially less inflammatory than TNFα or IL1β.

**Supplemental Figure 4. Raw ECIS data for hCMVEC barrier formation.** The ECIS data is presented as non-normalised resistance (ohms), which reveals the stable formation of the barrier within 24 hours of seeding. Typically the resistance achieved was constantly in the magnitude of >900-1000 ohms when using the 96W20idf ECIS arrays.
